# Comorbidities of HIV infection and health care seeking behavior among HIV infected patients attending public sector healthcare facilities in KwaZulu-Natal: A cross sectional study

**DOI:** 10.1371/journal.pone.0170983

**Published:** 2017-02-02

**Authors:** Manimbulu Nlooto

**Affiliations:** Discipline of Pharmaceutical Sciences, School of Health Sciences, University of KwaZulu- Natal, Durban, South Africa; Temple University School of Medicine, UNITED STATES

## Abstract

**Background:**

HIV-infected people may present with co-infections, comorbidities, and side effects associated with antiretroviral therapy. This study explored the prevalence of comorbid health problems and determined the extent of the use of traditional medicine for treatment of co-infections, comorbidities of HIV infection and side effects.

**Methods:**

A cross sectional study, using researcher-administered questionnaires, was carried out among HIV-infected patients in eight public sector healthcare facilities in KwaZulu-Natal between April and October 1024. Self-reports of comorbidities, co-infections and side effects were analyzed with respect to factors such as age, gender, race, and health care seeking behavior including the use of traditional medicine. Cross-tabulations were conducted to test the association between factors and the use of traditional medicine, using Pearson chi-squared (χ2) test. Simple and multiple logistic regression models tested the association of the use of traditional medicine with age, gender, race, side effects and comorbidities. Odds ratios with 95% confidence intervals were estimated. Missing values were handled, defined and treated as missing values in the final analysis.

**Results:**

Overall, 29.5% (n = 516) of the survey participants reported having other comorbidities and or co-infections besides their HIV condition. Same participants reported two or more comorbidities. Almost forty percent of participants (208/531, 39.17%) reported having hypertension as the most noninfectious comorbidity while 21.65% of participants (115/531) had tuberculosis accounting for the most infectious comorbidity. Almost eight percent of participants (142/1748, 8.12%) reported using traditional medicine after starting with cART. Sixty out of 142 participants (60/142, 42.25%) on cART resorted to the use of traditional medicine for the management of comorbidities and or co-infections of their HIV infection. Overall, 311 out of 1748 participants (17.80%) complained of ARVs related side-effects. Forty-five percent of those with side-effects (141/311, 45.34%) reported taking various types of medicines for treating side-effects, with 90.07% of them (127/141) using medicines prescribed by biomedically trained doctors or by pharmacy personnel as over-the -counter medicines, p <0. 001. Very few participants (14/141, 9.93%) resorted to the use of traditional medicine for treating side effects associated with antiretroviral therapy with no significant difference (p=0.293). In a multiple logistic regression, after adjusting for age, gender, race and side-effects due to antiretroviral therapy, odds for using traditional medicine were almost two times higher [odds ratio = 1.884, 95% Confidence Interval 1.317–2.695] with those participants having comorbidities and co-infections, with a significant difference p-value< 0.001.

**Conclusions:**

Comorbidities, co-infections and side effects are prevalent among HIV-infected patients attending public sector healthcare facilities. Odds of using traditional medicine were almost two times higher and significantly associated with the presence of comorbidities and co-infections than for other factors. The presence of such comorbid health problems does not explain the increased use of traditional medicine among HIV-infected patients on antiretroviral therapy. Findings from this study should be interpreted cautiously as they cannot be generalized to the entire population of HIV-infected patients in KwaZulu-Natal. Studies on safety and efficacy of herbal traditional medicines are needed for beneficiation of the minority of patients who still resort to them for co-treatment with combination antiretroviral therapy.

## Introduction

The burden of human immunodeficiency virus (HIV) infection is high in sub-Saharan Africa. In 2014, there were an estimated 25.8 million people living with HIV infection in the region [1]. South Africa has the largest HIV epidemic in the world with 6.8 million people living with HIV and an estimated prevalence rate of 18.9% among adults aged 15 to 49 years [2]. The KwaZulu-Natal province had the highest prevalence rate in South Africa with 16.9% [3]. Worldwide many people have taken or are currently taking complementary and alternative medicine (CAM) in combination with prescribed antiretroviral medicines [4]. Traditional medical practices can include the use of animal, mineral-based medicines, massages, spiritual therapies and a variety of other techniques unique to different regions and cultures [5]. African traditional health practitioners and those practicing other forms of traditional medicine (TM) not indigenous to African communities are an important point of access to health care for many Africans; they provide information, counselling and treatment options to members of the community [6].

Many HIV-infected patients first use TM before attempting western medicine and some use traditional medication in conjunction with western medicine due to illiteracy [7]. In South Africa, up to 90% of people living with human immunodeficiency virus (HIV) infection and acquired immunodeficiency syndrome (AIDS) use the services of a traditional healer before consulting biomedically trained healthcare providers [8]. Thus, the practice of traditional healing and other forms of alternative medicines are a health care resource in the South African society.

HIV–infected individuals may present with side effects due to combination antiretroviral therapy (cART) and/or co-infections and comorbidities preexisting before starting on or developing because of cART. Comorbidity may have a prolonged effect on health outcomes of HIV–infected individuals and their survival [9]. Other studies have previously reported the presence of comorbidities either infectious or non-infectious such as cardiovascular diseases, diabetes and others among individuals on cART [10, 11].

The potential risk associated with cART and development of metabolic syndrome in sub-Saharan Africa has been demonstrated in another study [12]. Anecdotal evidence from South Africa suggests that a number of ART patients resort to traditional medicine after experiencing side effects from ART [13]. Furthermore, the number of people with HIV infection on cART is increasing [14]. However, it is not clear whether the presence of co-infections and comorbidities of HIV infection influences health care seeking behavior among HIV-infected patients on ART. We hypothesized that co-infections, comorbidities, and adverse events led to a medical pluralism including the use of traditional medicine.

The concomitant use of ART and herbal medicines has been a matter of concern with the risk of pharmacokinetic or pharmacodynamics herbal-drug interaction [15]. Evidence of renal damage in HIV-infected patients with high CD4 cell counts has been demonstrated following the use of traditional medicine [16]. Some patients may present many challenges such as delays in visiting biomedically trained healthcare workers and healthcare facilities at the last stage of their disease, as they would not have been aware of their illness and status because of first consulting traditional health practitioners (THPs) [17]. Healthcare professionals caring for HIV-infected patients should be aware of the potential of herb-drug interactions and screen their patients for concomitant use of prescribed antiretroviral medicines (ARVs) and traditional medicine. Although HIV–infected have free access to cART, the use of traditional medicine is still prevalent among HIV-infected patients attending public sector healthcare facilities in parts of KwaZulu-Natal [18]. Building on the above previous works, this study sought to understand in a high HIV prevalence context the association between the presence of comorbidities, co-infections and side effects and health care seeking behavior including the use of traditional medicine among HIV infected individuals on cART. This study explored the prevalence of self-reported co-infections and comorbidities of HIV infection on one hand and the presence of ART associated adverse events on the other hand. The aim of the study was to determine whether HIV-infected patients used traditional medicine for the treatment of co-infections, comorbidities of HIV infection and adverse events after starting with cART in public sector healthcare facilities in Durban and Ladysmith areas, KwaZulu-Natal, South Africa.

## Materials and methods

### Sampling and selection of clinics

**Fig 1:**
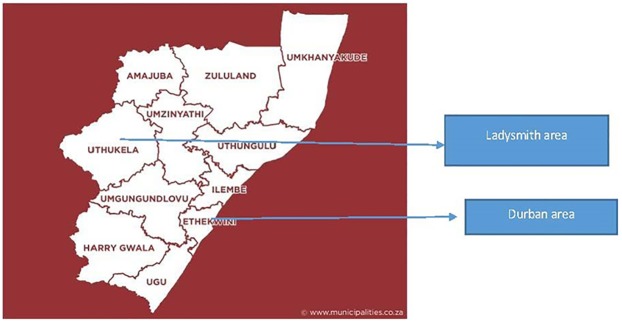
Map of Study Area.

From a list of 28 previously accredited public sector antiretroviral sites (outpatient clinics) which included 20 clinics in the Durban area which includes Ethekwini Metropolitan Health District and 8 clinics in the Ladysmith area, Uthukela district health district, KwaZulu-Natal, South Africa, a 50% stratified random sample from each district was taken. That resulted in 10 clinics from the Ethekwini Metropolitan Health District being selected and 4 clinics from the Uthukela health district. All 14 health clinics were approached and invited to participate in the survey. All invited clinics were divided into four levels of care (Community Health Centre, district hospital, regional hospital and tertiary hospital). However, permission to enter their premises was obtained from only eight healthcare facility managers who were gatekeepers at the time of data collection between April and October 2014. One clinic in Uthukela and seven clinics in EThekwini agreed to participate. One clinic served a rural population and seven served urban and peri-urban populations. No demographic data were available for the clinics that declined participation, so it was not possible to compare and contrast them with the clinics that agreed to participate. However, all 14 approached clinics, those who agreed and those who did not, served a population which was predominantly Black African. Fig 1. shows the study sites.

### Study design

A descriptive cross-sectional survey was conducted, using a researcher administered semi-structured questionnaire.

### Sampling and selection of participants

#### Study population

Study participants consisted of HIV infected patients, irrespective of gender, who were on antiretroviral treatment for three months and above. Three months were selected as the interval to allow side effects to have occurred.

#### Procedure for recruitment and selection of participants

Participants were selected at antiretroviral sites during clinic visit hours over a period of two to three weeks within the data collection time between April and October 2014. Every second patient entering the premises of the surveyed healthcare facility between April and October 2014 was approached to participate. If a patient declined participation, the second patient after that person was approached. This continued until the desired sample size of 1,766 was reached.

#### Inclusion and exclusion criteria of study participants

Eligible participants aged 18 and above were included in this study. Only patients coming in for clinic visits were approached for participation in the study, thus participants included in the study were those engaged in the healthcare system during the time of the study.

Patients less than 18 years old and that not on antiretroviral treatment and or on antiretroviral treatment but for less than three months were excluded. Participants who were defaulting or lost to follow-up were not included. Health care professionals caring for HIV infected patients in the surveyed districts were excluded from this study.

#### Sample size and assumptions

The study sample size was calculated using the formula, *n* = *Z*^2^(1-α/2) *pq*/*d*^2^ (where *Z* = 1.96 at 95% confidence interval, α or 5% error probability [type 1]); *p* = proportion of patients concurrently utilizing antiretroviral treatment with traditional medicine, *q* = 1-*p*; *d* = absolute allowable error (precision around the proportion you are wanting to estimate i.e. how wide the 95% confidence interval. A maximum variability was assumed, hence *p* = 0.5; *q* = 0.5 with a desired precision (*d)* ±5%. This yielded a minimum sample size of 384. To maintain this precision across the four levels of care described above and to detect at least 10% difference in health seeking behavior including the concurrent use of traditional medicines and prescribed antiretroviral therapy, the maximum required sample size was 384*4 = 1,536, irrespective of gender [19, 20]. To account for dropouts and unforeseen this minimum sample was oversampled by 15% to include a maximum sample size of 1766 participants.

### Data collection and variables

The research team members consisting of six final year pharmacy students, two retired nurses and two pharmacists, conducted a face-to-face administered interview, using a semi-structured questionnaire with mixed close-ended and open-ended questions. Interviews were meant to last for a maximum of 20 minutes. They were administered on the premises of surveyed clinics. Questionnaires gathered information on demographics, co-infections, comorbidities, side effects and health care seeking behavior including the use of traditional medicine. Questionnaires were administered in English and in isiZulu depending on respondent preference. They consisted of three sections: section A solicited socio-demographic data of respondents; section B collected clinical information about the prescribed antiretroviral treatment and other medications; and section C was used to assess health seeking behavior including the use of traditional medicine before and after starting with cART.

#### Variables

Socio-demographic characteristics of study participants, co-infections, comorbidities and side effects were self–reports from study participants. Study participants were asked a set of questions specifically developed for this study. They were asked if they had other illnesses besides their HIV infection, side effects due to their cART, what types of antiretroviral medicines (ARVs) they were on. Other questions probed treatment modalities used by study participants in their health care seeking behavior, including the use of traditional healers, for management of comorbidities, co-infections and side effects. Response options were yes and no to the question if they had other illnesses besides their HIV infection and or side effects. If yes, they were further asked to list such illnesses, side effects experienced and treatment options, including categories of traditional healers visited. Researchers have not administered ARVs to participants; however, ARVs listed by study participants were those prescribed according to the South African national antiretroviral guidelines 2014, which consisted mainly of a fixed dose combination (FDC) in a single tablet containing a combination of tenofovir (TDF), emtricitabine (FTC) and efavirenz (EFV) [21]. Self-reports of health care seeking behavior of study participants, including use of traditional medicine, were collected and analyzed for self-management of comorbidities, co-infections and side- effects.

### Data analysis

All data was reviewed and checked for duplication. Descriptive statistics including percentages and proportions were used for overall characteristics of study participants and to determine the prevalence rate of co-infections, comorbidities, side effects and the use of traditional medicine among participants. Cross tabulations were performed. Association in contingency tables was assessed in the Pearson chi-squared (χ2) test to compare association between the presence of co-infections, comorbidities or side effects and the use of TM before and after starting with cART. The level of significance was estimated using a p value ≤ 0.05. Simple and multiple logistic regression models were later built to test the association of variables such as age, gender, race, side-effects and comorbidities with the use of traditional medicine as part of health care seeking behavior among study participants. Odds ratios (OR) with 95% confidence intervals (CI) were estimated. Cases with missing values were handled, defined and expressed as frequency and or percentage, where applicable. The only available data were treated in the final analysis. No specific methods such as imputations were done to treat missing data that were in a small number. A p-value ≤0.05 was estimated as statistically significant.

### Ethical considerations

This study received ethical clearance from the University of KwaZulu-Natal, Biomedical Research Ethics Committee (BREC), under reference number BE 377/13. Permission to conduct the study was obtained from the KwaZulu-Natal Department of Health. An information letter was given or read to eligible participants. Those willing to participate were requested to sign an informed consent form, also available in isiZulu, before being interviewed. No names and identity of participants appeared on any consent form except a signature and a coded identification known to the researchers. No patients were included in this research study without their prior signed consent form.

## Results and discussion

### Socio-demographic characteristics of participants

This study enrolled 1,748 participants out of a targeted maximum of 1,766 participants, yielding 98.98% response rate.

Table 1 presents a set of socio-demographic characteristics of the study participants previously reported by Nlooto & Naidoo, 2016 (18). Overall, two thirds of study participants were Black African (1685/1748, 96.40%) and 73.80% (1290/1748) were female. The median age of participants was 36–40 years (range 18–66). The majority of participants had no formal income (1030/1748, 58.92).

**Table 1:** Socio-demographic characteristics of participants.

Category	Sub-category	(N, %)
Getnder	Female	1290 (73.80)
	Male	458 (26.20)
	Total	1748(100)
Race	Black African	1685(96.40)
	Coloured	39(2.23)
	Indian	17(0.97)
	White	4(0.23)
	Missing	3(0.17)
	Total	1748(100)
Age in years	Median age group	36-40(range18-65)
	18–20	73 (4.18)
	21–25	74 (4.23)
	26–30	179 (10.24)
	31–35	336 (19.22)
	36–40	377 (21.57)
	41–45	303 (17.33)
	46–50	187 (10.70)
	51–55	131 (7.49)
	56–60	72 (4.12)
	61–65	2 (0.11)
	Missing	9 (0.51)
	Total	1748 (100)
Income situation	No formal income	1030(59.82)
	≤R10000($8333.3)	585((33.47)
	R10100-200000($8334–16666)	86(4.92)
	R201000-300000($16666–25000)	12(0.69)
	R301000-400000($25000–33333	5(0.29)
	R 401000-500000($33333–41666)	2(0.11)
	Self-employed	4(0.22)
	Missing	23(1.32)
	Total	1748(100)

R: South African Rand

#### Characteristics of participants and frequency of comorbidities and co-infections

Table 2 presents the cross tabulations between characteristics of participants and comorbidities. Nearly a third of participants of the entire sample (516/1748, 29.5%) reported having comorbidities and co-infections besides their HIV condition. There was no significant difference between female and male participants with regard to frequency of comorbidities and co-infections, p = 0.413. Age and race were significantly associated with the presence of comorbidities and co-infections in the sample (p<0.05). Gender and income were not significantly associated with comorbidities and co-infections.

**Table 2:** Characteristics of participants and frequency of comorbidity.

Category	Sub-category	Frequency of comorbidities (N, %)			
		Yes	NO	Total	Pearson chi-squared (χ2) test
Gender	Female	388(30.08)	902(69.92)	1290(100)	0.413
	Male	128(27.95)	330(72.05)	458(100)	
	Total	516(29.52)	1232(61.48)	1748(100)	
Race	Black African	486(28.84)	1199(71.16)	1685(100)	0.011(<0.05)
	Coloured	14(35.90)	25(64.10)	39(100)	
	Indian	10(58.82)	7(41.18)	17(100)	
	White	4(100)	0	4(100)	
	Missing	2(66.7)	1(33.3)	3(100)	
	Total	516(29.52)	1232(70.48)	1748(100)	
Age in years	Median age group	36–40 (range 18–65)			
	18–20	42(57.53)	31(42.47)	73(100)	0.000(<0.001)
	21–25	10(13.51)	64(86.49)	74(100)	
	26–30	34(18.99)	145(81.01)	179(100)	
	31–35	59(17.56)	277(82.44)	336(100)	
	36–40	82(21.75)	295(78.25)	377(100)	
	41–45	91(30.03)	211(69.97)	303(100)	
	46–50	77(41.18)	110(58.82)	187(100)	
	51–55	74(56.49)	57(43.51)	131(100)	
	56–60	41(56.94)	31(43.06)	72(100)	
	61–65	0	2(100)	2(100)	
	Missing	1(11.11)	8(88.89)	9(100)	
	Total	516(29.52)	1232(70.48)	1748(100)	
Income	No formal income	314(30.49)	716(69.51)	1030(100)	0.324
	≤R10000($8333.3)	170(29.06)	415(70.95)	585(100)	
	R10100-200000($8334–16666)	13(15.12)	73(84.88)	86(100)	
	R201000-300000($16666–25000)	3(25)	9(75)	129100)	
	R301000-400000($25000–33333)	2(40)	3(60)	5(100)	
	R401000-500000($33333–41666)	1(50)	1(50)	2(100)	
	Self-employed	1(25)	3(75)	4(100)	
	Missing	12(52.17)	11(47.83)	23(100)	
	Total	516(29.52)	1232(70.48)	1748(100)	

R: South African Rand

#### Types of Comorbidities and co-infections of HIV infection

Table 3 presents the frequency of types of comorbidities and co-infections reported by study participants. Overall, 29.52% of the participants (516/1748) reported having comorbidities, co-infections and other AIDS defining conditions. However, same participants reported two or more conditions. Comorbidities consisted mostly of hypertension (208/531, 39.17%) while co-infections were mainly tuberculosis infections (115/531, 21.65%).

**Table 3:** Comorbidities and co-infections reported by study participants.

Category	Sub-Category	Frequency	Percent (%)
Comorbidity	Hypertension	208	39.17
	Diabetes	66	12.43
	Asthma	28	5.27
	Epilepsy	28	5.27
	Renal Disease	11	2.07
	Heart disease	10	1.88
	Gastric ulcers	10	1.88
	Hypertension and Diabetes	10	1.88
	Subtotal	371	69.87
Co-infections	Tuberculosis	115	21.65
	Bacterial infections	8	1.51
	Herpes simplex	7	1.32
	Oral thrush or candidiasis	2	0.38
	Subtotal	132	24.86
AIDS defining conditions	Weight loss	9	1.69
Other*	Combined hypertension and tuberculosis	19	3.58
	Subtotal	28	5.27
Total	-	531	100

* Two or more conditions were reported by same participants.

A few participants reported having AIDS defining conditions such as weight loss (9/516, 1.74%). Other participants listed more than two comorbidities and or co-infections, which included amongst others: hypertension and diabetes (10/516, 1.94%), hypertension and tuberculosis (19/516, 3.68%).

### Self–reported prescribed antiretroviral medicines used by study participants

The majority of participants reported being on a FDC antiretroviral regimen (1140/1748, 65.20%), followed by multiple dose tenofovir containing regimens (426/1748, 24.37%), other non-identified regimens (122/1748, 6.98%), lopinavir/ritonavir containing regimens (24/1748, 1.37%), zidovudine containing regimens (22/1748, 1.26%) and stavudine containing regimens (15/1748, 0.86%).

### Self-reported side-effects of ART

Table 4 presents the side effects reported by patients. Overall, 311 out of 1748 participants complained of ARVs related side effects (17.80%). The mostly self-reported side effects were skin rashes (57/311, 18.33%), followed by painful and swollen feet (10.61%).

**Table 4:** Self- reported side-effects by study participants.

Category	(N, %)
Skin rashes	57(18.33)
Painful and swollen feet*	33(10.61)
Severe headaches	25(8.03)
Nausea and vomiting	22(7.07)
Weakness and fatigue	19(6.11)
Dizziness	16(5.14)
Weight loss	16(5.14)
Diarrhea	15(4.82)
Lipodystrophy	10(3.22)
Hallucinations and bad dreams	9(2.90)
Loss of appetite	7(2.25)
Increased sickness due to ART	4(1.29)
Gynecomastia	3(0,96)
Miscellaneous	39(12.54)
Unknown	36(11.58)
Missing	10(3.22)

ART = antiretroviral therapy,

* = more than one reported

### Health care seeking behavior for self-reported comorbidities / co-infections and side-effects

Table 5 presents a contingency table between the presence of comorbidities and co-infections and the use of traditional medicine after starting with cART. At least eight percent of participants (142/1748, 8.12%) reported using traditional medicine after starting with cART.

**Table 5:** Cross-tabulations of comorbidities/co-infections and use of traditional medicine after starting cART among study participants.

Comorbidity/co-infection	Use of TM after starting with cART			
	Yes (N, %)	No (N, %)	Total (N, %)	Pearson Chi squared test (χ2)
Yes	60 (11.63)	456 (88.37)	516 (100)	P<0.001
NO	81(6.60)	1146(93.40)	1227(100)	
Missing	1(20)	4(80)	5(100)	
Total	142 (8.12)	1606 (91.88)	1748(100)	

Before starting with cART 125 participants out of those with other illnesses (125/516,24.22%) reported using TM provided for treating comorbidities and co-infections, p = 0.115. After starting with cART, eleven percent of participants (60/516, 11.63%) used TM for the management of comorbidities and other co-infections. There was a significant reduction in the use of TM for comorbidities before (24.22%) and after (11.63%) starting with cART, p<0.001.

The majority of those who used concurrently TM with ARVs for comorbidities and co-infections visited abaprofeti or prophets and spiritual healers from African Independent Churches (14/60,23.3%), followed by inyangas or herbalists(13/60, 21.7%), sangomas or diviners(5/60,8.3%), front shop in a pharmacy (3/60,5%) and family members(1/60, 1.7%) while the other cases (24/60,40%) did not list the category of traditional healers that they visited.

Table 6 presents a contingency table between the presence of side effects and the use of traditional medicine. After handling missing values and treating them as missing. Nearly half of participants who had side- effects (141/311, 45.34%) reported taking various types of medicines for treating such side effects. The majority reported using prescribed medicines by biomedically trained doctors and by pharmacy personnel as over-the counter preparations at a pharmacy (127/141, 90.07%), p <0.001. Very few participants (14/141, 9.93%) resorted to the use of TM with no significant difference, p = 0.293.

**Table 6:** Presence of side effects and use of traditional medicine after starting with cART.

Any side-effects for taking ARVs	Use of TM after starting with cART			
	Yes (N, %)	No (N, %)	Total (N, %)	Pearson Chi squared test (χ2)
Yes	30 (9.65)	281 (90.35)	311 (100)	P = 0.293
NO	112 (7.84)	1136 (92.16)	1428 (100)	
Total	142 (8.17)	1597 (91.83)	1739 (100)	

#### Simple and multiple logistic regression analysis

In a simple logistic regression model after handling missing values and treating them as missing, associations were tested between age, gender, race, presence of comorbidities and side–effects and use of traditional medicine, assumed as a dependent variable. Odds of using traditional medicine were significantly associated with the presence of comorbidities and co-infections, p<0.001, OR = 1.839, 95% CI [1.296–2.610] while age (p = 0.413, OR = 1.038, 95% CI [0.949–1.135]), gender (p = 0.079, OR = 0.718,95% CI [0.497–1.039]), race (p = 0.371, OR = 0.687, 95% CI [0.302–1.563]) and the presence of side-effects (p = 0.294, OR = 1.254, 95% CI [0.822–1.915]) were not significantly associated with the use of traditional medicine.

#### Multivariate logistic regression analysis

Table 7 presents associations between variables and use of traditional medicine in a multiple logistic regression model. After handling missing values and treating them as missing, gender (p = 0.046, OR = 0.684, 95% CI [0.471–0.992]) and presence of comorbidities and co-infections (p = 0.001, OR = 1.884, 95% CI [1.317–2.695]) were significantly associated with the use of traditional medicine. The odds of using traditional medicine were almost two times high (OR = 1.884) with the presence of comorbidities and co-infections.

**Table 7:** Association between variables and use of traditional medicine.

Variable	Use of traditional medicine after starting cART(n = 142)		
	P-Value	Odds ratio	Odds ratio 95% CI
Age	0.802	1.011	0.926–1.105
Gender	0.046	0.684	0.471–0.992
Race	0.212	0.590	0.258–1.351
Comorbidities/co-infections	0.001	1.884	1.317–2.695
Side-effects	0.309	1.247	0.815–1.909

CI = confidence interval

Almost all the study participants (99.27%) believed that ART had helped them due to improvement in their quality of life and a decreased viral load and increased CD4 as they took their medication from month to month. Half of participants (874/1748, 50%) reported that they were told at pre-counseling sessions for getting onto ART not to use TM concurrently with their ARV medication. On follow up visits 882 participants (882/1748, 50.42%) reported being shunned for their use of TM by their healthcare provider at the clinic (Doctors, Nurses, Pharmacists and Counselors). Despite the negative attitudes of healthcare workers to participants using TM, a few participants (205/1748, 11.73%) believed that traditional healers should be given a chance to find a cure for HIV infection.

## Discussion

This study found a high burden of comorbidities such as hypertension and diabetes; co-infections with tuberculosis and side effects due to cART among HIV infected people attending public sector healthcare facilities in KwaZulu-Natal. The observed prevalence of comorbidities in this study is consistent with the report on the rate of comorbidities of HIV infection in sub-Saharan Africa [12]. A similar study conducted in Canada showed that comorbidities are prevalent among HIV infected individuals on cART [22]. This study found that comorbidities were mainly chronic diseases affecting the cardiovascular (hypertension, heart disease) and endocrine (diabetes) systems. This finding is in agreement with the premature age-related comorbidities in a cohort of HIV patients in Italy [10]. Comorbidity of HIV infection in sub-Saharan Africa has been associated with negative physical functioning among patients receiving antiretroviral therapy [23]. HIV infected people with fewer comorbid health problems were reported to have better physical functioning in KwaZulu-Natal, South Africa [24]. We found that a few participants had more than two comorbidities and co-infections. This is in agreement with a study conducted in Tanzania where the presence of two or more opportunistic infections were reported among HIV infected people [25].

This study found that age and racial groups were associated with significant levels of comorbidities and co-infections. In a cross-sectional population, based study measuring comorbidity among people living with HIV in Canada age was shown to be associated with medical comorbidity [22]. Although female participants in this study were predominant in the breakdown of the sample included; there was no significant difference found in the association between female and male participants with regard to the frequency of comorbidities and co-infections(p = 0.413). However, in a multivariate logistic regression analysis, gender was significantly associated with the use of traditional medicine after starting with cART, p = 0.046, OR = 0.684 95%CI 0.471–0. 992. We cannot draw firm conclusions with this association. In another study among HIV infected patients with oral lesions in Tanzania, no statistically significant association was found with socio-demographic features, including gender (p = 0.68) [26]. In a systematic review, Littlewood & Vanable (2008) concluded that many studies were inconclusive on the role of gender and age in predicting the use of complementary and alternative medicine [27].

This study found that a proportion of participants had tuberculosis coinfections and or other infections such as herpes simplex or oral thrush. In Malaysia, the most commonly found comorbidities were infectious diseases such as hepatitis C and other opportunistic infections [28]. Tuberculosis coinfections may worsen the quality of life among HIV-infected patients [23]. In addition, participants in this study reported side effects due to cART. The presence of side effects has raised concerns because side effects can have negative limiting effects on benefits of cART. Boyer et al. [29] reported that side effects have been associated with lower scores on the physical and mental health scales among HIV infected people. However, this study differed from previous works in this way that it focused not only on determining the prevalence of comorbidities, co-infections of HIV infection and side-effects due to cART, but also it looked at associations between certain factors and health care seeking behavior among HIV-infected individuals experiencing such comorbidities.

Although the role of traditional medicine has been demonstrated in the management of chronic diseases [30], it is speculated from other another that the use of traditional medicine has declined among HIV infected patients on antiretroviral therapy in South Africa [18]. A study conducted among HIV infected patients in KwaZulu-Natal demonstrated a decrease in the use of traditional medicine during follow-up visits to antiretroviral clinics [31]. This is probably because of the counselling given to patients by healthcare workers before starting cART and subsequent attitudes of healthcare workers towards patients using traditional medicine. This study found that the odds of using traditional medicine was almost two times high among HIV infected patients on cART and with comorbidities and or co-infections. A few participants in this study have stated using traditional medicine for comorbid health problems hence healthcare professionals should screen patients seen by them for potential risks of herb-drug interactions [32]. In addition, safety and efficacy of herbs used in co-treatment of HIV infection is not well-documented [33]. The presence of comorbidities and co-infections of HIV infection may lead to seek for options such as herbal therapies, which are believed to improve the well-being among HIV infected individuals [34]. In a study carried out in Zimbabwe, prevention and treatment of illnesses were reported as the main motivating factors for using traditional medicine among adults [35]. The perception that traditional healers have an approach to health which offers information, counselling and treatment options to members of the community [6] may be another explanation for the increased use of traditional medicine among HIV individuals with comorbidities.

This study found no association between side- effects of cART and use of traditional medicine by study participants but rather they resorted to prescribed medicines by biomedically trained or dispensed by pharmacy personnel as over- the-counter medicines. This finding is in contrast with another study, which concluded that treatment of side effects related to conventional antiretroviral treatments was one of the motivations for using complementary and alternative medicine among people living with HIV or AIDS [36]. This finding further disagrees with the anecdotal evidence from South Africa suggests that a number of ART patients resort to traditional medicine after experiencing side effects from ART [13].

### Limitations of the study

Although this study included no defaulters and lost to follow-up participants but rather active participants attending public sector health care facilities, it is difficult to make a causal inference between the use of traditional medicine and the presence of comorbidities and co-infections among study participants. However, with regard to the power of the sample size, this study can serve to generate a hypothesis that comorbidities and co-infections of HIV infection may lead patients on cART to an increased use of traditional medicine. Given that the study was conducted in two health districts, the findings should be interpreted with caution, as they cannot be generalized to the entire population of HIV-infected patients in KwaZulu-Natal in particular and South Africa in general. As previously discussed by Levin (2006), results may differ within another timeframe and settings [37]. Other studies could be replicated in settings that are more rural.

## Conclusions

This study found that comorbidities, coinfections and side effects are prevalent among HIV infected attending public sector healthcare facilities. A few participants on cART still resorted to the use of traditional medicine for the management of comorbidities and coinfections of HIV infection, and side effects due to antiretroviral therapy. Odds of using traditional medicine were almost two times higher and significantly associated with the presence of comorbidities and co-infections than for other factors.

Nearly half of participants who had side- effects reported taking various types of medicines for treating side-effects, with the majority reporting using prescribed medicines by biomedically trained doctors and by pharmacy personnel as over-the counter preparations at a pharmacy, p <0.001. Caution is advised in the interpretation of the findings of this study. Further studies in more rural settings are needed. Studies on safety and efficacy of herbal traditional medicines are also needed for health benefits to the patients who still resort to them for co-treatment with cART.

## Supporting information

S1 Publication Nlooto M, Naidoo P(2016). (PDF)

S1 File Final Book1for analysis combined tshim-neeri-nlooto revised November 2015.xlsx. (XLSX)

S2 File Code book combined tshim-neeri-nlooto.xlsx. (XLSX)
