# Supplementary material for: Comorbidities of HIV infection and health care seeking behavior among HIV infected patients attending public sector healthcare facilities in KwaZulu-Natal: A cross sectional study
Source: PLoS One. 2017 Feb 2;12(2):e0170983. doi: 10.1371/journal.pone.0170983 (PMC5289501; doi:10.1371/journal.pone.0170983)
Supplement: S1 Publication — (PDF) [file pone.0170983.s001.pdf]

Nlooto and Naidoo *BMC Complementary and Alternative Medicine* (2016) 16:128
DOI 10.1186/s12906-016-1101-5

BMC Complementary and Alternative Medicine

RESEARCH ARTICLE Open Access

Traditional, complementary and alternative medicine use by HIV patients a decade after public sector antiretroviral therapy roll out in South Africa: a cross sectional study

Manimbulu Nlooto* and Panjasaram Naidoo

Abstract

Background: The roll out of antiretroviral therapy in the South African public health sector in 2004 was preceded by the politicisation of HIV-infection which was used to promote traditional medicine for the management of HIV/AIDS. One decade has passed since; however, questions remain on the extent of the use of traditional, complementary and alternative medicine (TCAM) by HIV-infected patients. This study therefore aimed at investigating the prevalence of the use of African traditional medicine (ATM), complementary and alternative medicines (CAM) by adult patients in the eThekwini and UThukela Health Districts, South Africa.

Methods: A cross- sectional study was carried out at 8 public health sector antiretroviral clinics using interviewer-administered semi-structured questionnaires. These were completed from April to October 2014 by adult patients who had been on antiretroviral therapy (ART) for at least three months. Use of TCAM by patients was analysed by descriptive statistics using frequency and percentages with standard error. Where the associated relative error was equal or greater to 0.50, the percentage was rejected as unstable. A *p*-value <0.05 was estimated as statistically significant.

Results: The majority of the 1748 participants were Black Africans (1685/1748, 96.40 %, SE: 0.00045), followed by Coloured (39/1748, 2.23 %, SE: 0.02364), Indian (17/1748, 0.97 %, SE: 0.02377), and Whites (4/1748, 0.23 %, SE: 0.02324), $p < 0.05$. The prevalence of ATM use varied prior to (382/1748, 21.85 %) and after ART initiation (142/1748, 8.12 %), $p < 0.05$, specifically by Black African females both before (14.41 %) and after uptake (5.49 %), $p < 0.05$. Overall, 35 Black Africans, one Coloured and one Indian (37/1748, 2.12 %) reported visiting CAM practitioners for their HIV condition and related symptoms post ART.

Conclusion: Despite a progressive implementation of a successful antiretroviral programme over the first decade of free antiretroviral therapy in the South African public health sector, the use of TCAM is still prevalent amongst a small percentage of HIV infected patients attending public healthcare sector antiretroviral clinics. Further research is needed to explore reasons for use and health benefits or risks experienced by the minority that uses both conventional antiretroviral therapy with TCAM.

Keywords: African Traditional, Complementary, Alternative Medicine, HIV, Antiretroviral therapy, Prevalence

* Correspondence: Nlooto@ukzn.ac.za
Discipline of Pharmaceutical Sciences, School of Health Sciences, University of KwaZulu-Natal, Private Bag X 54001, Durban, South Africa

BioMed Central

© 2016 Nlooto and Naidoo. **Open Access** This article is distributed under the terms of the Creative Commons Attribution 4.0 International License (http://creativecommons.org/licenses/by/4.0/), which permits unrestricted use, distribution, and reproduction in any medium, provided you give appropriate credit to the original author(s) and the source, provide a link to the Creative Commons license, and indicate if changes were made. The Creative Commons Public Domain Dedication waiver (http://creativecommons.org/publicdomain/zero/1.0/) applies to the data made available in this article, unless otherwise stated.

Background

Human Immunodeficiency Virus (HIV) and Acquired Immunodeficiency Syndromes (AIDS) are amongst the biggest health challenges faced by African nations. The last decade (2001–2010) saw many developing countries, including South Africa, roll out successful antiretroviral (ARV) programmes through a global advocacy initiative to reduce the associated morbidity and mortality as well as the cost of ARV medicines to overcome the health care human resource crisis, to simplify the drug regimens, monitor and improve the quality of care [1]. Globally people tend to use traditional medicine under the terms complementary and alternative medicines (CAM), including for HIV and other chronic conditions [2]. HIV-infected patients may turn for their health management to public, private, household/self-care and other community based non-formal health sectors such as traditional and faith healers, herbalists and other vendors [3]. In South Africa, public sector healthcare is offered to patients with HIV-infection at different levels of the health care system: Primary Health Care (PHC) clinics, Community Health Centres (CHC), District Hospitals (DH), Regional Hospitals (RH), Academic Hospitals (AH) and Central Hospitals (CH).

South Africa launched its first *Operational Plan for Comprehensive HIV and AIDS Care, Management and Treatment* with its national antiretroviral guidelines in 2004. During that time, HIV was highly politicised in the country, with very senior officials denying the link between HIV and AIDS; the latter was seen as a condition associated with poverty in the socio-economic contexts of South Africa [4]. The politicisation of HIV and AIDS was also used before 2004 to promote traditional, complementary and alternative medicines (TCAM) by senior government officials who advocated the use of treatment modalities outside the orthodox system of HIV treatment, such as lemon juice, beetroot and garlic [5]. The period after the introduction of highly active antiretroviral therapy (HAART) has been marked by the political will towards promoting universal access to antiretroviral therapy (ART) as well as integrating traditional medicine into the public health care sector [6]. The political support has resulted in South Africa having one of the most successful public health sector ARV programmes on the African continent, with at least two million people receiving ART [7]. One critical question remains however on the prevalence of TCAM use a decade after easy access to ARV medicines was made possible by the government.

The World Health Organisation (WHO) defines African traditional medicine (ATM) as *"The sum total of all knowledge and practices, whether explicable or not, used in the diagnosis, prevention and elimination of physical, mental, or societal imbalance, and relying exclusively on practical experience and observation handed down from generation to generation, whether verbally or in writing"* [8]. Other forms of health care, such as Ayurveda and Traditional Chinese medicine, which are not part of Africa's own traditional medicine category, are not integrated into the dominant health care systems, but fall under CAM [9]. The practice of African traditional healing in South Africa is regulated by the Traditional Health Practitioners Act (Act 35 of 2004, further replaced by Act 27 of 2007) while the practice of CAM is legally allowed under the Allied Health Practitioners Act (Act 63 of 1982) and includes Ayurveda, Traditional Chinese Medicine, Osteopathy, Chiropractic, Homeopathy, Naturopathy, Phytotherapy, Therapeutic Aromatherapy, Massage therapy, and Reflexology [10]. Some HIV infected patients who receive care at public health sector ARV facilities may also use other methods of healing/treatment, either indigenous to African communities or not; the prevalence and reasons for their use are not well known.

South Africa has a diverse population made up of four main ethnic groups, namely Black African (79 %), White (9.6 %), Coloured (8.9 %) and Indian (2.5 %) [11]. However, the use of TCAM in combination with prescribed ARVs by HIV patients from different racial groups is not well known in the era of a successful ARV programme. The use of TCAM by HIV –infected patients in KwaZulu-Natal has been reported in the literature [12–14]. Data from one such study conducted in KwaZulu-Natal showed that TCAM was commonly used for HIV- infection during the 6 months study period (317/638, 51.3 %) in combination with prescribed ART [15]. Concerns relating to the safety of uncontrolled medicinal products have been raised with reference to intrinsic toxicity and quality issues associated with TCAM; other risks include adverse drug reactions such as liver toxicity reportedly associated with the use of kava-kava [16] and drug interaction with ARVs and medicinal plants [17]. Another concern is a delay reported in starting with ART amongst HIV patients who use the services of traditional healers in pre-ART phase in Mozambique [18] whilst issues with HIV patients on ART abandoning treatment for exclusively being managed by prayers and faith healing were noted in Zambia [19]. Poor adherence to ART was also noted with the concurrent use of TCAM and ARVs [20]. Although studies reported poor adherence in HIV patients on concurrent use of TCAM and ART [21, 22], another study documented that patients adhering to ART for HIV/AIDS also used CAM measures such as homeopathy and herbal medicines for ARVs side-effects, immune system enhancement, and overall well-being [23]. This study therefore aimed at determining whether HIV patients attending public health sector ARV clinics still used TCAM for HIV-infection while on a successful ARV programme. The specific objectives were to determine the prevalence of the use of TCAM for HIV –infection pre –ART and its ongoing use after starting with

ARVs. In addition the study was conducted to ascertain whether socio-economic status and/or the apartheid structure of the past had an influence on the use of TCAM by HIV infected patients.

Methods

Study design and sites

A descriptive cross sectional study was conducted from April to October 2014 using a questionnaire with both closed-ended and open-ended questions administered through face to face interviews to HIV- infected patients attending public healthcare sector ARV clinics. Fourteen ARV clinics at different levels of care were approached to be part of the study, but permission was obtained from eight clinics situated in the eThekwini and uThukela health districts. The ARV clinics were from three regional hospitals (RH), one academic hospital (AH), two district hospitals (DH), and two community health centres (CHC). Study sites in eThekwini consisted of three ARV clinics located in previously designated Black African communities, two ARV clinics situated in a previously designated Coloured community, and a previously designated Indian community, whilst another clinic was in an informal urban settlement and the last site in Ethekwini Metro was in a previously White designated area. The ARV site in UThukela health district was located in a town, previously designated for the White community.

All of the 8 clinics were mainly in the metropolitan and urban types of settlement in both sampled health districts [24]. The South African National Department of Health (NDOH) paid a rural allowance to healthcare professionals depending on the type of locality and place of health facilities; some metropolitan settlements have those types of locality qualifying for rural allowance like in normal rural areas. The description of ARV clinics by the designator 'with or without allowance' was used in this analysis to distinguish the type of locality and place of clinics. For anonymity and confidentiality the eight ARV sites were coded according to their designated level as: RHRA1, RHRA2, RHNRA3, AHNRA4, DHRA5, DHNRA6, CHCRA7 and CHCNRA8, with the suffixes RA and NRA referring to rural and non-rural allowance paid to health care professionals, respectively and the prefixes were the type of healthcare facility, for example, RH (Regional hospital), AH (.Academic hospital), DH (District hospital) and CHC (Community health centre).

Study sample size assumption

A sample size was calculated using a formula previously described in the literature [25, 26]. The assumption for the calculation of the sample size (n) was based on an expected prevalence (P) of 50 % on the use of TCAM among study participants, with a precision (d) of ±5 % and a level of confidence interval at 95 %. This yielded a minimum sample size of 384. This study was conducted at four levels of care (academic, regional, district hospitals and community health centres). To maintain this precision across antiretroviral sites per level of care, the minimum sample size was multiplied by four and was estimated at 384*4 = 1536 participants. To allow for drop –outs and unforeseen circumstances, the sample size was oversampled by 15 % for a maximum of 1766 participants, irrespective of gender. But for logistic reasons, time constraints and willingness of participants to be part of the study during data collection, 1748 adult volunteers were recruited and included in this analysis.

Ethical approval

This study was approved by the Biomedical Research Ethics Committee (BREC) of the University of KwaZulu-Natal; under reference number BE 377/13. Participation was voluntary; no patients were included in this research study without their prior consent. No names and identity of participants appeared on any consent form, but only a signature and a coded identification known to the researchers. All questionnaires were coded to ensure anonymity and confidentiality. Questionnaire readily available in English and Zulu allowed a choice of a preferred language by study participants. Field workers, who collected data, were briefed about the study and the importance of maintaining confidentiality and anonymity of the study participants.

Recruitment process and selection of participants

Study participants were all HIV infected adult patients, aged 18 years and above, irrespective of gender. Adult patients visiting the ARV clinic on the days of data collection and who satisfied the inclusion criteria were eligible to participate in the study. Participants were approached when entering the premises of the ARV clinic and were asked if they would like to participate in the study. An information sheet/letter about the study was given or explained to them. Those who agreed to participate were requested to give and sign consent before being interviewed. Only patients who were on HAART for at least 3 months were included in the final analysis.

Data collection technique and instrument

A semi-structured questionnaire with both close- and open-ended questions was used to conduct face to face interviews by a team of trained field workers. Interviews were conducted once off, face to face by one member of the research team, in the language of preference of the study participants, which was either Zulu or English. The questionnaire included amongst others socio-demographic characteristics of participants and a section on the use of TCAM for the management of HIV-infection prior to and after starting with ART. For this

analysis one such question stated" Have you ever used TCAM for the management of HIV-infection prior to and after starting with ART? If yes, please list the category of traditional healer visited for your HIV condition before and after starting with ARVs." Other sections of the questionnaire related to reasons and factors associated with use of TCAM for HIV and non –HIV related problems, and or perceived benefits/risks of TCAM; the results of the latter have not been included in this paper.

Statistical analysis

Data captured on Excel sheet was managed and analysed using the Software Package for Social Sciences (SPSS), version 22 for Windows; SPSS Inc,2014–2016 (Chicago,IL). Results were presented as frequency and percentages with standard error (SE). Where the associated relative error was equal or greater to 0.50, the percentage was rejected as unstable. Categorical variables were presented as frequency with tables or graphs. Pearson chi-square was performed for most of the variables; with exceptions of certain cases where Fischer's Exact or McNemar tests were more appropriate to determine the level of significance. Bivariate analyses were used to assess socio-demographic characteristics in relation to the use of ATM pre ART as well as ATM and CAM use post ART. The influence of socio-demographic characteristics on the odds of using ATM and CAM for HIV infection was estimated for each characteristic by a multivariable logistic regression including 95 % confidence intervals for odds ratio and p –values. A p-value < 0.05 or better was estimated as statistically significant.

Results

Response rate

Face to face interviews were completed with 1748 respondents out of the targeted maximum sample of 1766 participants, yielding a response rate of 98.98 %.

Socio- demographic characteristics of the study population

Participants were predominantly Black Africans (1685/1748, 96.40 %, SE: 0.00045), followed by Coloured (2.23 %, SE: 0.02364), then by Indian (0.97 %, SE: 0.02377) and negligible White participants (0.24 %, SE: 0.02324), p –value < 0.05. Participants were mostly Zulu speakers (87.93 %, SE:0.00831), Christians (77.00 %, SE:0.01148), never married (75.74 %, SE:0.01178), females (73.80 %, SE:0.01224), aged between 31 and 45 years old (58.12 %, range 18–66), had high school education (1259/1748, 72.03 %), were unemployed and had no formal income (58.98 %, SE:0.01532) while the majority (33.47 %) of those with income made less than R100 000 per annum (US $1 = ZA R12 at the time of data collection). Socio-demographic characteristics are presented in Table 1.

Table 1 Socio-demographic characteristics of study participants (n = 1748), 2014

Variable	Category	No.	%	Standard error
Gender	Male	458	26.20	0.02055
	Female	1290	73.80	0.01224
Age in years	18–30	282	16.13	0.02351
	31–65	1442	82.49	0.02340
	≥66	15	0.86	0.02148
	Missing value	9	0.51	0.02374
Racial group	African	1685	96.40	0.00454
	Coloured	39	2.23	0.02364
	Indian	17	0.97	0.02377
	White	4	0.23	0.02324
	Missing value	3	0.17	0.02366
Marital status	Single/never married	1324	75.74	0.01178
	Married	319	18.25	0.02162
	Divorced	21	1.20	0.02376
	Living with partner	59	3.38	0.02353
	Widow	19	1.09	0.02382
	Missing value	6	0.34	0.02376
Home language	Zulu	1537	87.93	0.00831
	Other African languages	77	4.40	0.02340
	Xhosa, other SA languages	70	4.00	0.02342
	English	57	3.26	0.02352
	Afrikaans	2	0.11	0.02343
	Urdhu	1	0.05	0.02449
	Missing value	4	0.22	0.02395
Highest education level	Primary school	332	18.99	0.02277
	High school	1259	72.03	0.02335
	Post high school diploma	40	2.29	0.02365
	Higher certificate	82	4.69	0.02335
	Bachelor degree	13	0.74	0.02377
	Never attended school	15	0.86	0.02384
	Missing value	7	0.40	0.02386
Income situation in thousands	Unemployed/ no formal income	1031	58.98	0.01532
	≤ R100 000 ($8333.3)	585	33.47	0.01951
	8333–16666.6)	86	4.92	0.02332
	16750–25000)	12	0.69	0.02390
	25083–33333.3)	5	0.29	0.02405
	33417–41667)	2	0.11	0.02344
		4	0.22	0.02342

Table 1 Socio-demographic characteristics of study participants ($n = 1748$), 2014 *(Continued)*

	Self-employed & undisclosed income			
	Missing value	23	1.32	0.02380
Religious affiliation	Christians	1401	80.14	0.01148
	Shembe	140	8.00	0.02294
	Zion Christian Church (ZCC)	97	5.54	0.02325
	Atheists	54	3.09	0.02355
	Believers in ancestors/ traditional Zulu	31	1.77	0.02368
	Muslims	14	0.80	0.02381
	Hindus	7	0.40	0.02386
	Missing value	4	0.22	0.02395

Legend: *SA*= South African

Prevalence of the use of TCAM for HIV-infection pre and post ART

There was a decrease in ATM use for HIV infection post ART by a total of 13.73 % from 382 (21.85 %) to 142 participants (8.12 %), p-value < 0.05. A few participants (37/1748, 2.12 %) reported the use of CAM post ART for HIV infection ($p = 0.920$). Table 2 presents the frequency of the use of ATM and CAM pre and post ART by study participants.

Prior to ART 382 out of 1748 participants (21.85 %) reported ATM use for the management of HIV infection. Majority of attendees of public sector healthcare ARV clinics were Black Africans who also reported more the use of TCAM for HIV prior to and post ART than those participants from the other three racial groups. Very few Coloured (3/1748), Indian (2/1748) and White participants (1/1748) agreed to have used ATM for HIV prior to ART compared to the majority of Black Africans (376/1748). Overall 37 participants including 35 Black Africans, one Coloured and one Indian (37/1748, 2.12 %) reported CAM use. None of the few White participants continued with ATM and or resorted to CAM use post ART. The use of CAM (37/1748, 2.12 %) was lower than ATM use (142/1748, 8.12 %) for HIV infection in the post ART phase.

There were more Zulu speakers than other categories of home languages who resorted to ATM prior to (336/1748, 19.22 %) and after (125/1748, 7.15 %). Single participants have used more ATM than other categories of marital status prior to (285/1748, 16.30 %) and after ART (106/1748, 6.06 %), $p < 0.05$. Almost all age groups in this study used ATM healers prior to and post ART, suggesting that age was a constant variable with a relatively high proportion of users aged between 31 and 50 years old prior to (268/1748, 15.33 %) and after ART (102/1748, 5.84 %), p-value < 0.05.

Almost none of the few participants (19/1748,1.08 %) with income between R 201000-300000 ($16750–41667) had ever used ATM prior to and after ART. Being unemployed with no formal income was associated with a high proportion of participants' use of ATM prior to (178/1748, 10.18 %) and post ART (74/1748, 4.23 %), with a significant difference ($p < 0.05$.). Participants with high school education (101/1748, 5.78 %) used more ATM healers and CAM practitioners (27/1748, 1.54 %) than those with primary and post- secondary education.

Frequency of the use of TCAM by income category, level of education and religious affiliations is presented in Table 3.

The majority of ATM users and those who consulted CAM practitioners were Christians. No Hindu participant reported using ATM or CAM practitioners pre and post ART whilst very few Muslims (5/1748) used ATM for HIV pre –ART. Comparisons of sample characteristics and the use of ATM healers prior to and after ART were performed using Chi-square tests.

Female participants used more ATM prior to (252/1748, 14.41 %) and post ART (96/1748, 5.49 %) than males; many more women resorted post ART to the services of CAM practitioners for HIV (29/1748) than males (8/1748) in both types of locality and levels of care of clinics. Gender differences in the use of ATM prior to and post ART (p-value < 0.05) are illustrated in Fig. 1.

Current utilization of ARV clinics by study participants per types of locality and levels of care of clinics

In spite of inclusion of four ARV clinics situated in areas previously designated for the other three racial groups, current utilization of ARV clinics was mostly and actually dominated in both types and locality of clinics by Black African attendees.

The majority of participants were from clinics 'without rural allowance' coded NRA as suffix (1111/1748, 63.56 %) vs clinics 'with rural allowance' and coded RA as suffix (637/1748, 36.44 %), p-value <0.05. Table 4 presents the current utilization of clinics.

Use of TCAM by type of locality and level of care of ARV clinics by study participants

Prior to ART this study found as many participants from clinics without rural allowance (193/1748, 11.04 %) who used ATM healers as from clinics with rural allowance (189/1748, 10.81 %). However; post ART, there were many more participants from clinics without rural allowance (89/1748, 5.09 %) who used some types of ATM in combination with prescribed ARVs than those from clinics with rural allowance (53/1748, 5.03 %), p-value < 0.05. Overall, participants attending district hospitals used more ATM post ART (89/1748, 7.95 %) than those attending community health centres (31/1748, 1.77 %),

Table 2 ATM and CAM use by gender, racial group, age and home language, 2014

Variable	Total sample (*N* = 1748)	ATM use prior to ART (*n* = 382)	ATM use post ART (*n* = 142)	Chi-square *P*-value[a]	CAM use post ART (*n* = 37)	*P*-value [b]
Gender						
Male	458	130	46	0.000	8	0.791
Female	1290	252	96	0.000	29	
Racial group						
Black	1685	377	139	0.000 [c]	35	0.920
Coloured	39	3	2	0.000 [d]	1	
Indian	17	1	1	0.000	1	
White	4	1	0	0.000	0.	
Age (years)						
18–19	29	5	1	0.029	0	0.373
20–25	74	17	5	0.000	1	
26–30	179	39	13	0.000	6	
31–35	337	74	23	0.000	6	
36–40	374	70	28	0.000	10	
41–45	305	78	33	0.000	4	
46–50	187	46	18	0.000	3	
51–55	132	20	4	0.000	5	
56–60	77	22	11	0.000	0	
61–65	30	9	4	0.001	2	
≥66	15	2	2	0.000	0	
Marital status						
Single	1324	285	106	0.000	27	0.018
Married	319	67	26	0.000	6	
Divorced	21	3	1	0.012	0	
Living with partner	59	26	9	0.001	4	
Widow	19	1	0	NA	0	
Home language						
Zulu	1537	336	125	0.000	34	0.000
Other African languages	77	6	4	0.000	1	
Xhosa and other South African languages	70	18	4	0.000	1	
English	57	21	9	0.000	0	
Afrikaans	2	0	0	*NA*	0	
Urdhu	1	1	0	*NA*	1	

ART antiretroviral therapy, *ATM* African traditional medicine, *NA* not applicable
Legend: [a:] Cross tabulations and Chi-square tests were performed; [b] Non-parametric tests were performed; [c] Pearson Chi-square, [d]Fisher's Exact

regional and academic hospitals combined (22/1748, 1.26 %), *p*-value < 0.05. This suggests the role of services of healthcare facilities in the decrease of the use of ATM healers and CAM practitioners. Figure 2 illustrates the use of TCAM by type of locality and level of care of clinics prior to and post ART.

Prevalence of the use of CAM practitioners was higher in ARV clinics with rural allowance (21/1748,1.20 %) than in those clinics without rural allowance (16/1748,0.92 %), *p*-value <0.05. The use of ATM prior to and post ART varied significantly per ARV clinic/health facility ($p = 0.000$) and type of locality of ARV clinic with or without rural allowance ($p = 0.000$). level of care Post ART the use of CAM per level of care ($p = 0.000$) and location of ARV clinics ($p = 0.010$) was statistically significant.

Table 3 Frequency of ATM and CAM use by income category, level of education and religion ($n = 1748$)

Highest education level	Total sample ($N = 1748$)	ATM use prior to ART ($n = 382$)	ATM use post ART ($n = 142$)	Chi-square *P*-value [a]	CAM use post ART ($n = 37$)	*p*-value[b]
Some primary school	163	48	18	0.000	3	0.421
Completed primary school	169	44	14	0.000	3	
Some high school grade 7 to grade 11	700	156	54	0.000	18	
Completed high school	559	109	47	0.000	9	
Post high school diploma	40	3	0	*NA*	1	
Higher certificate	82	14	5	0.000	1	
Bachelor degree	13	3	2	0.005	1	
Never attended school and other	15	4	2	0.035	1	
Missing value	7	1	0	*NA*	0	
Income situation						
Unemployed and no formal income	1031	178	74	0.000	19	0.274
≤R 100 000 ($8333.3)	585	159	60	0.000	16	
8333–16666.6)	86	37	4	0.030	1	
16750–25000)	12	1	0	*NA*	0	
25083–33333.3)	5	0	0	*NA*	1	
33417–41667)	2	0	0	*NA*	0	
Self-employed and undisclosed income	4	2	2	0.135	0	
Missing value	23	5	2	0.046	0	
Religious affiliation						
Christian	1345	249	89	0.000	24	0.725
Shembe	140	47	18	0.000	6	
Zion Christian Church (ZCC)	97	36	23	0.000	4	
Atheist	54	8	2	0.000	0	
Believers in ancestors/ traditional Zulu	31	18	4	0.000	1	
Muslims	14	5	0	*NA*	0	
Hindus	7	0	0	*NA*	0	
Other (St Johns,Chibihi, Wisile)	56	18	6	0.000	2	
Missing value	4	1	0	*NA*	0	

ART antiretroviral therapy, *ATM* African traditional medicine, *NA* not applicable
Legend: [a:] Cross tabulations and Chi-square tests were performed; [b] Non parametric tests were performed

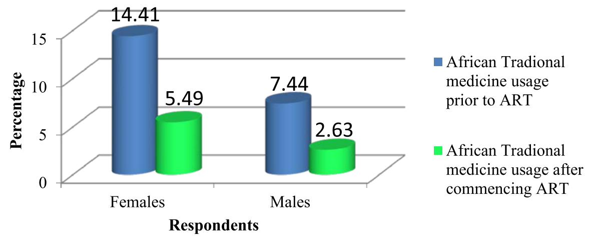

Fig. 1 Gender differences in the use of African traditional medicine prior and after joining antiretroviral programmes, 2014

Bivariate and multivariate analysis of use of ATM and CAM by participants

Bivariate analyses indicated that having an income category ≤ R 100,000 (US$ 8,333.3 at the time of data collection) and being Christian was associated with the use of ATM post ART while conversely no socio-demographic characteristic was significantly associated with CAM use post ART. Female gender, being Black and Christian for religious affiliation, having completed high school, post-secondary higher certificate, diploma and degree, gaining an income of ≤ R 100,000 and ≥100,000 (US $ 8,333.3) were

Table 4 Types of locality of clinics and utilization of clinics by racial groups (n=1748), 2014

Facility	Type of locality	Previous racial designation	Current utilization of clinics by participants by racial group	Number (%)
RHRA1	Township	Black	African	111 (6.35)
			Subtotal	111 (6.35)
RHRA2	Urban	White	African	100 (5.72)
			White	1 (0.06)
			Missing	1 (0.06)
			Subtotal	102 (5.84)
RHNRA3	Township	Indian	African	102 (5.84)
			Coloured	1 (0.06)
			Indian	7 (0.40)
			Subtotal	110 (6.30)
AHNRA4	Urban	White	African	336 (19.22)
			Coloured	2 (0.12)
			Indian	3 (0.17)
			Subtotal	341 (19.51)
DHRA5	Rural	Black	African	279 (15.96)
			Coloured	1 (0.06)
			Indian	1 (0.06)
			Missing	1 (0.06)
			Subtotal	282 (16.14)
DHNRA6	Urban	Coloured	African	381 (21.80)
			Coloured	33 (1.89)
			Indian	6 (0.34)
			White	3 (0.17)
			Missing	1 (0.06)
			Subtotal	424 (24.26)
CHCRA7	Township	Black	African	142 (8.13)
			Subtotal	142 (8.13)
CHCNRA8	Informal urban		African	234 (13.39)
			Coloured	2 (0.12)
			Subtotal	236 (13.51)

Legend: *AH* academic hospital, *CHC* community health centre, *DH* district hospital, *RH* regional hospital, *NRA* non- rural allowance

significantly associated with ATM use prior to ART (see Table 5 below).

In the multivariate logistic regression being Christian was confirmed to be associated with the use of ATM for HIV infection post ART (OR 0.46, 95 % CI 0.32–0.66, p-value < 0.001). The lack of association of socio-demographic characteristics with CAM use post ART was confirmed in the multivariate analysis. However, female gender, being black and Christian for religious affiliation, having completed high school, post-secondary higher certificate, diploma and degree, and gaining income were confirmed in the multivariate model as associated with the use of ATM prior to ART. Table 5 presents the bivariate and multivariate analyses for the use of ATM and CAM by study participants.

Discussion

This study found that a few HIV patients still used ART concurrently with TCAM for their HIV-infection a decade after the implementation of a successful ARV programme in South Africa. However there was a decrease on the use of TCAM evidenced by a drop in the percentage of participants that took TCAM prior to (21.85 %) and post ART (8.12 %). This finding is consistent with data obtained from other studies. In one such study conducted between October 2007 and February 2008 in KwaZulu-Natal the authors reported a decline in prevalence of complementary or traditional treatments in HIV-infected patients from 36.6 % prior to ART to 7.9 % after 6 months on ART [12]. In another longitudinal follow-up study the authors reported a further decrease in TCAM at 12 and 20 months post ART [27].

The utilization of public sector ARV clinics in this study was predominantly by Black African females with the majority reporting being single and never married. Many more females used ATM healers and visited CAM practitioners in the post ART era than males as evidenced in this study. The lower percentage of males visiting ARVclinics could be supported by another study that found that men reported several barriers to the use of public and private clinics for sexual health services in Gauteng province (South Africa), and many preferred the services of traditional healers for sexually –transmitted –infections [28]. Another study found an increased use of CAM amongst women in rural areas in Australia [29].

Almost none of the participants in the relatively high income bracket/category had used ATM healers or CAM practitioners post ART, this is in contrast with findings of another study done amongst African-Americans where the high income category afforded participants to use CAM for treatment [30]. However, the finding of this study was consistent with the findings from a national household survey on the utilization of traditional healers in South Africa which showed that the wealthiest had a tendency neither to consult traditional healers nor to use public sector healthcare facilities for health issues [31]. This may justify the high number of Black African HIV patients attending public sector ARV clinics, with a few Coloured, Indian and White participants also not reporting a substantial use of TCAM.

The role played by the type of dwelling and the the participants' place of residence place and locality of clinics has been demonstrated by patients in seeking

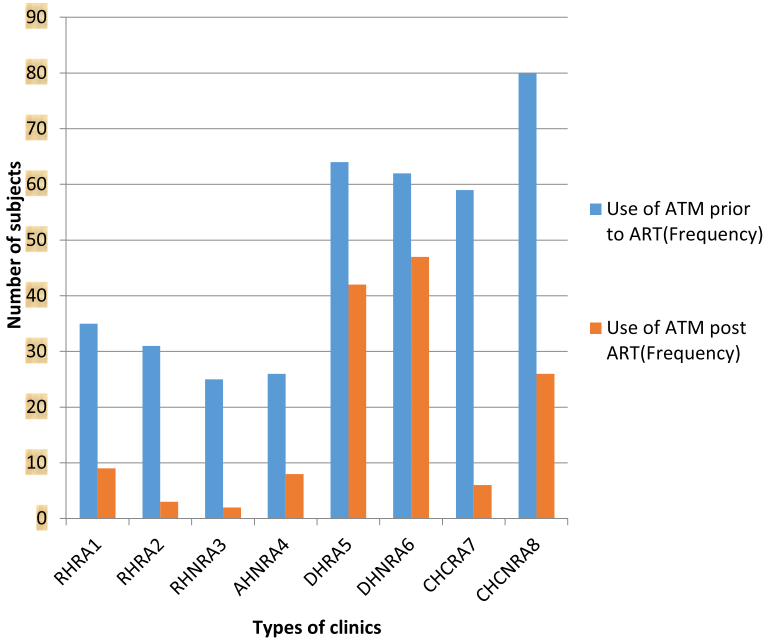

Fig. 2 Use of ATM by type of locality and level of care of clinics, 2014

care after traditional healers [32]. We found that there was no significant difference between the types of locality and place of clinics and the use of ATM prior to ART, but there was a significant decline in the prevalence of the use of ATM healers and CAM practitioners after joining the ARV programme. The use of TCAM in the pre-ART phase may have been due to the unaffordability and inaccessibility of HAART. People had to find alternate means to manage their HIV condition. A few participants in this study visited CAM practitioners for HIV infection (37/1748, 2.12 %) when compared to ATM healers (142/1748, 8.12 %). Patterns of CAM use and ethnic-specific CAM use vary across racial/ethnic groups demonstrating the role of culture in choices of the use of CAM practitioners [33].

From this study and other previous studies we can hypothesize that the introduction and progressive free access to ART in public sector healthcare facilities have contributed to the decline in the use of TCAM amongst South African HIV infected patients a decade after the roll-out of ARV programme. Challenges of pharmacological interactions caused by concomitant use of traditional herbal therapies and conventional antiretroviral medicines should be a cause of concern for quality of care offered to patients and should be investigated further for risk of clinically significant pharmacokinetic (PK) interactions [17].

Since a low percentage of HIV patients in this study still used TCAM for HIV-infection, further studies are needed to determine reasons and factors associated with the use of TCAM, and or benefits/risks of concurrent use of TCAM with ART while on a successful programme.

Strengths and limitations of the study

This study included a reasonable sample size and looked at the diverse population of South Africa. The response rate was very satisfactory to meet expectations of more or less 80 %, with acceptable reliability and validity for this type of research [34]. The percentage of missing value for almost all variables in this study was very low, if not negligible. As part of limitations there might be a recall bias which could have affected the categories of ATM healers and CAM practitioners reported by participants. This study discussed the use of ATM differentiated to CAM use as defined in the context of South Africa. This study looked at public sector healthcare ARV programme; no attempt was made to understand the phenomenon in the private sector healthcare system. The study sample was exclusively made of patients attending public health sector ARV clinics and it did not include the general population; therefore findings cannot be generalized to the entire population of persons living with HIV infection in the study sites or South Africa. The study was conducted during clinic visit hours, not allowing participants full freedom of expression, which could have influenced the participants' response on the use of TCAM.

Conclusions

A decade after the introduction of ART and despite the success of the public health sector ARV programme in

Table 5 Odds ratios (OR) and 95 % confidence intervals (CI) for ATM and CAM use, bivariate and multivariate analyses

Variable	Use of ATM prior to ART(yes = 382,No = 1365)				Use of ATM post ART(Yes = 142, No = 1602)				Use of CAM post ART(Yes = 37, No = 1710)			
	Bivariate OR (95 % CI)	p-value	Multivariable OR (95 % CI)	p-value	Bivariate OR (95 % CI)	P-value	Multivariate OR (95 % CI)	p-value	Bivariate OR (95 % CI)	p-value	Multivariable OR (95 % CI)	p-value
Gender												
Male	1 (ref)		1 (ref)		1 (ref)		1 (ref)		1 (ref)		1 (ref)	
Female	0.61 (0.48,0.78)	<0.001	0.69 (0.53,0.89)	0.005	0.72 (0.5,1.04)	0.079	0.76 (0.52,1.11)	0.152	1.11 (0.52,2.37)	0.788	1.17 (0.54,2.54)	0.683
Race												
other	1 (ref)		1 (ref)	0.005	1 (ref)		1 (ref)		1 (ref)		1 (ref)	
Black	3.17 (1.26,7.98)	0.014	3.87 (1.43,10.49)	0.008	1.71 (0.53,5.54)	0.369	1.91 (0.53,6.84)	0.323	1.29 (0.17,9.57)	0.804	0.75 (0.08,7.44)	0.806
Age												
≤ 30	1 (ref)		1 (ref)		1 (ref)		1 (ref)		1 (ref)		1 (ref)	
> 30	1 (0.75,1.33)	0.985	0.88 (0.65,1.2)	0.427	1.09 (0.7,1.72)	0.693	1.02 (0.64,1.6)	0.947	0.72 (0.33,1.53)	0.389	0.66 (0.31,1.43)	0.293
Marital status												
Not in union	1 (ref)		1 (ref)		1 (ref)		1 (ref)		1 (ref)		1 (ref)	
Married or living with partner	1 (1,1)	0.766	1 (1,1)	0.776	1 (1,1)	0.453	1 (1,1)	0.258	1.34 (0.64,2.8)	0.431	1 (0.96,1.04)	0.868
Mother tongue												
Non-Zulu	1 (ref)		1 (ref)		1 (ref)		1 (ref)		1 (ref)		1 (ref)	
Zulu	0.98 (0.69,1.39)	0.91	0.71 (0.48,1.05)	0.089	0.99 (0.58,1.68)	0.977	0.83 (0.47,1.49)	0.539	1.54 (0.47,5.06)	0.477	1.62 (0.41,6.32)	0.488
Education	Bivariate OR (95 % CI)	p-value	Multivariable OR (95 % CI)	p-value	Bivariate OR (95 % CI)	P-value	Multivariate OR (95 % CI)	p-value	Bivariate OR (95 % CI)	p-value	Multivariable OR (95 % CI)	p-value
Primary	1 (ref)		1 (ref)		1 (ref)		1 (ref)		1 (ref)		1 (ref)	
Some high school	0.75 (0.55,1.01)	0.057	0.74 (0.54,1.02)	0.064	0.79 (0.5,1.24)	0.303	0.83 (0.52,1.34)	0.45	1.44 (0.56,3.65)	0.447	1.41 (0.55,3.62)	0.480
Grade 12	0.63 (0.46,0.87)	0.005	0.61 (0.43,0.86)	0.005	0.86 (0.54,1.38)	0.543	0.92 (0.56,1.52)	0.739	0.89 (0.31,2.53)	0.828	0.85 (0.29,2.48)	0.762
Higher certificate, diploma, degree	0.5 (0.3,0.82)	0.006	0.46 (0.27,0.78)	0.004	0.6 (0.28,1.29)	0.189	0.67 (0.31,1.47)	0.318	1.49 (0.41,5.35)	0.542	1.49 (0.4,5.48)	0.551
Income												
None	1 (ref)		1 (ref)		1 (ref)		1 (ref)		1 (ref)		1 (ref)	
≤ R100 000	1.78 (1.4,2.28)	<0.001	1.92 (1.49,2.49)	<0.001	1.48 (1.03,2.11)	0.032	1.44 (0.99,2.09)	0.054	1.49 (0.76,2.93)	0.243	1.58 (0.79,3.16)	0.199
> R 100 000	2.73 (1.79,4.16)	<0.001	3.38 (2.18,5.25)	<0.001	0.75 (0.32,1.77)	0.512	0.85 (0.36,2.03)	0.714	0.98 (0.23,4.28)	0.982	1.07 (0.24,4.77)	0.925
Religion												
Other	1 (ref)	<0.001	1 (ref)	<0.001	1 (ref)		1 (ref)		1 (ref)		1 (ref)	
Christian	0.46 (0.36,0.59)	<0.001	0.49 (0.37,0.64)	<0.001	0.46 (0.32,0.66)	<0.001	0.52 (0.35,0.75)	<0.001	0.54 (0.27,1.07)	0.076	0.55 (0.27,1.1)	0.092

South Africa, the use of TCAM is still prevalent amongst a small percentage of HIV infected patients attending public sector healthcare ARV clinics. In the pre-ART phase, many patients tend to use TCAM. However, there is a decrease in the use of TCAM in the post ART phase. An hypothesis can be assumed that free access to ART may account in the decrease of the use of TCAM by HIV patients. Public sector healthcare ARV clinics are mainly used by Black African patients, with a few patients from the other racial groups. Although the majority of patients did not report using TCAM while on ART, further research is needed to explore reasons for use and health benefits or risks experienced by the minority that uses both conventional antiretroviral therapy and traditional medicine.

Ethics approval and consent to participate

This study was approved by the Biomedical Research Ethics Committee (BREC) of the University of KwaZulu-Natal; under reference number BE 377/13. Participation was voluntary; no patients were included in this research study without their prior consent

Consent for publication

Not applicable in this section.

Availability of data and materials

The authors do not wish to share publicly the database at this stage; other manuscripts emanating from this data are underway.

Abbreviations

AH: Academic Hospital; AIDS: Acquired immunodeficiency syndrome; ART: Antiretroviral therapy; ARV: Antiretroviral; ATM: African traditional medicine; BREC: Biomedical research ethics committee; CAM: Complementary and alternative medicines; CH: Central Hospital; CHC: Community Health Centre; DH: District Hospital; HAART: Highly active antiretroviral therapy; HIV: Human immunodeficiency virus; NA: Not applicable; NRA: Non- rural allowance; PHC: Primary Health Care Clinic; RA: Rural allowance; RH: Regional Hospital; SE: Standard error; TCAM: Traditional, complementary and alternative medicines; WHO: World Health Organisation; ZA R: South African Rand; ZCC: Zion Christian Church.

Competing interests

The authors declare that they have no competing interests.

Authors' contributions

MN and PN conceptualized and designed the study; MN organized data collection, analyzed, interpreted data and drafted the paper. PN revised the manuscript critically for important intellectual content. The two authors gave final approval of the version to be published.

Authors' information

The authors choose not to use this section.

Acknowledgements

We thank the College of Health Sciences and DST-NRF Excellence Centre, Indigenous Knowledge Systems, University of Kwa Zulu-Natal for their support. Many thanks to Jamillah Karrem, Sarah Bata, Amanda Nhlengethwa, Nokuthula Cynthia Radebe, Nonkulelo Felicity Dhlamini, and Sinegugu Claire Jali for their efforts in the collection of data. We are grateful to Ms Carrim Martin for her editing services. Big thanks to Professor Benn Sartorius for volunteering to review the statistical analysis of this study.

"Open access publication of this article has been made possible through support from the Victor Daitz Information Gateway, an initiative of the Victor Daitz Foundation and the University of KwaZulu-Natal."

Funding

MN received a stipend from DST-NRF and running expenses from the College of Health Sciences of the University of KwaZulu-Natal for his doctoral research.

Received: 29 October 2015 Accepted: 21 April 2016
Published online: 17 May 2016

References

1. Ford N, Calmy A, Mills EJ. The first decade of antiretroviral therapy in Africa. Global Health. 2011;7:33.
2. Bodeker G, Ong CK, Grundy C, Burdford G, Shein K. World Health Organization, Global atlas of traditional, complementary and alternative medicine: Text volume. Geneva: WHO Press; 2005. Available at: apps.who.int/bookorders/MDIbookPDF/Book/11500614.pdf Accessed 03 January 2016
3. Moshabela M, Pronyk P, Williams N., Schneider H, Lurie M. Patterns and implications of medical pluralism among HIV/AIDS patients in rural South Africa. *AIDS Behav*. 2011; 15: 842–52 Available at www.ncbi.nlm.nih.gov/pubmed/20628898 Accessed 03 January 2016
4. Mbali M. AIDS Discourses and the South African State: Government denialism and post-apartheid AIDS policy-making. Transformation. 2004;54:104–22. Available at http://kznhass-history.net/ojs/index.php/jnzh. Accessed 13 January 2016.
5. Chigwedere P, Seage 3rd GR, Gruskin S, Lee TH, Essex M. Estimating the lost benefits of antiretroviral drug use in South Africa. J Acquir Immune Defic Syndr. 2008;49(4):410–5.
6. Grant M, Haskins L, Gaede B, Hornwood C. Bridging the gap: exploring the attitudes and beliefs of nurses and patients about coexisting traditional and biomedical healthcare systems in a rural setting in KwaZulu-Natal. S Afr Fam Pract. 2013;55(2):175–9. Available at http://www.ajol.info/index.php/safp/article/view/88391. Accessed 7 Jan 2016.
7. Venter F, HIV Treatment in South Africa. The challenges of an increasingly successful antiretroviral programme, South African Health Review. Durban: Health Systems Trust; 2013. Available at https://www.health-e.org.za/wp-content/uploads/2013/04/SAHR2012_13_lowres_1.pdf. Accessed 14 Dec 2015.
8. World Health Organization. Traditional Medicine Strategy 2002–2005. Geneva: World Health Organization; 2002. Available at http://www.wpro.who.int/health_technology/book_who_traditional_medicine_strategy_2002_2005.pdf. Accessed 03 Dec 2015.
9. World Health Organization: Traditional Medicine Growing Needs and Potential - WHO Policy Perspectives on Medicines, No. 002, May 2002. Available at http://apps.who.int/medicinedocs/en/d/Js2293e/. Accessed 3 Dec 2015
10. Gqaleni N, Indres M, Kruger H, Ntuli A, McLeod H. Traditional and complementary medicine. In South African Health Review.2007. Durban: Health Systems Trust; 2007. Available at http://www.healthlink.org.za/uploads/files/SAHR2007.pdf Accessed 03 Jan 2016
11. Statistics South Africa. Statistical release P0302 Mid-year Population Estimates 2011. Pretoria: Statistics South Africa; 2011. Available at http://www.statssa.gov.za/publications/P0302/P03022011.pdf. Accessed 03 Jan 2016.
12. Peltzer K, Friend-du Preez N, Ramlagan S, Fomundam H, Anderson J. Traditional, complementary and alternative medicine and antiretroviral treatment adherence among HIV patients in KwaZulu-Natal, South Africa. Afr J Tradit Complement Altern Med. 2010;7(2):125–37.
13. Hughes GD, Puoane TR, Clark BL, Wondwossen TL, Johnson Q, Folk W. Prevalence and predictors of traditional medicine utilization among persons living with AIDS (PLWA) on antiretroviral (ARV) and prophylaxis treatment in both rural and urban areas in South Africa. Afr J Tradit Complement Altern Med. 2012;9(4):470–84.
14. Babb DA, Pemba L, Seatlanyane P, Charalambous S, Churchyard GJ, Grant AD. Use of traditional medicine by HIV-infected individuals in South Africa in the era of antiretroviral therapy. Psychol Health Med. 2007;12(3):314–20.
15. Peltzer K, Friend Du Preez N, Ramlagan S, Fomundam H. Use of traditional, complementary and alternative medicine for HIV patients in KwaZulu-Natal, South Africa. BMC Public Health. 2008;8:255.
16. Kayne SB, Traditional Medicine. A Global Perspective. 1st ed. London: Pharmaceutical Press; 2009. Available at: http://www.dandybooksellers.com/acatalog/9780853698333.pdf. Accessed 03 January 2016.

17. Müller AC, Kanfer I. Potential pharmacokinetic interactions between antiretrovirals and medicinal plants used as complementary and African traditional medicines. Biopharm Drug Dispos. 2011;32(8):458–70.
18. Audet CM, Salato J, Blevins M, Amsalem D, Vermund SH, Gaspar F. Educational intervention increased referrals to allopathic care by traditional healers in three high HIV-prevalence Rural Districts in Mozambique. PLoS One. 2013;8(8):1–8.
19. Musheke M, Bond V, Merten S. Self-care practices and experiences of people living with HIV not receiving antiretroviral therapy in an urban community of Lusaka, Zambia: implications for HIV treatment programmes. AIDS Res Ther. 2013;10(1):12.
20. Owen-Smith A, Diclemente R, Wingood G. Complementary and alternative medicine use decreases adherence to HAART in HIV-positive women. AIDS Care. 2007;19(5):589–93.
21. Tamuno I. Traditional medicine for HIV infected patients in antiretroviral therapy in a tertiary hospital in Kano; Northwest Nigeria. Asian Pac J Trop Med. 2011;4(2):152–5.
22. Lubinga SJ, Kintu A, Atuhaire J, Asiimwe S. Concomitant herbal medicine and Antiretroviral Therapy (ART) use among HIV patients in Western Uganda: a cross-sectional analysis of magnitude and patterns of use, associated factors and impact on ART adherence. AIDS Care. 2012;24(11):1375–83.
23. Hughes GD, Mbamalu ON. Integarative medicine in resource constrained communities in South Africa: Embracing indigenous Knowledge and traditional (herbal) medicine practice. Revista da ABPN. 2015;7(16):214–35. Available at http://abpn.org.br/Revista/index.php/edicoes/article/viewArticle/533. Accessed 17 December 2015.
24. Statistics South Africa. Census 2011. Available at http://www.statssa.gov.za/publications/P03014/P030142011.pdf. Accessed 20 August 2015.
25. Schork MA, Williams GW. Number of observations required for the comparison of two correlated proportions: Number of observations required for the comparison. Commun Statistics-Simulation Comput. 1980; 9(4):349–57.
26. Machin D, Campbell MJ, Tan SB, Tan SH. Sample size tables for clinical studies. Chichester: John & Wiley; 2011.
27. Peltzer K, Friend-du PN, Ramlagan S, Fomundam H, Anderson J, Chanesta L. Antiretrovirals and use the use of traditional, complementary and alternative medicine by HIV patients in KwaZulu-Natal, South Africa: a longitudinal study. Afr J Tradit Complement Altern Med. 2011;8(4):337–45.
28. Leichliter JS, Paz-Bailey G, Friedman AL, Habel MA, Vezi A, Sello M, et al. 'Clinics aren't meant for men': sexual health care access and seeking behaviours among men in Gauteng province, South Africa. SAHARA J. 2011;8(2):82–8.
29. Meurk C, Broom A, Adams J, Sibbritt D. Rurality, mobility, identity: women's use of complementary and alternative medicine in rural Australia. Health Place. 2013;20:75–80.
30. Barner JC, Bohman TM, Brown CM, Richards KM. Use of complementary and alternative medicine for treatment among African-Americans: a multivariate analysis. Res Social Adm Pharm. 2010;6(3):189–208.
31. Nxumalo N, Alaba O, Harris B, Chersich M, Goudge J. Utilization of traditional healers in South Africa and costs to patients: findings from a national household survey. J Public Health Policy. 2011;32 Suppl 1:S124–36.
32. Sangare AD, Samba M, Bourgeois D. Illness-related behaviour and sociodemographic determinants of oral health care use in Dabou, Côte d'Ivoire. Community Dent Health. 2012;29(1):78–84. Community Dent health, 2012 Mar; **29**(1):78–84.
33. Hsiao AF, Wong MD, Goldstein MS, Yu HJ, Andersen RM, Brown ER, et al. Variation in complementary and alternative medicine (CAM) use across racial/ethnic groups and the development of ethnic-specific measures of CAM use. J Altern Complement Med. 2006;12(3):281–90.
34. Fincham JE. Response rates and responsiveness for surveys, standards, and the Journal. Am J Pharm Educ. 2008;72(2):43.

Submit your next manuscript to BioMed Central and we will help you at every step:

- We accept pre-submission inquiries
- Our selector tool helps you to find the most relevant journal
- We provide round the clock customer support
- Convenient online submission
- Thorough peer review
- Inclusion in PubMed and all major indexing services
- Maximum visibility for your research

Submit your manuscript at www.biomedcentral.com/submit

BioMed Central
